# Automatic genetic phenotype normalization from dysmorphology physical examinations: an overview of the BioCreative VIII—Task 3 competition

**DOI:** 10.1093/database/baaf051

**Published:** 2025-09-24

**Authors:** Davy Weissenbacher, Xinwei Zhao, Jessica R C Priestley, Katherine M Szigety, Sarah F Schmidt, Karen O'Connor, Ekin Soysal, Kirk Roberts, Jiewei Qi, Ling Luo, Zhihao Yang, Hongfei Lin, Hajung Kim, Chanhwi Kim, Jiwoong Sohn, Tim Beck, Marek Rei, Sunkyu Kim, T Ian Simpson, Joram M Posma, Antoine Lain, Mujeen Sung, Jaewoo Kang, Areej Alhassan, Viktor Schlegel, Monira Aloud, Riza Batista-Navarro, Goran Nenadic, Ying-Jia Lin, Zhi-Quan Feng, Hung-Yu Kao, Ian M Campbell, Graciela Gonzalez-Hernandez

**Affiliations:** Cedars-Sinai Medical Center, 700 N. San Vicente Blvd. Pacific Design Center, West Hollywood, CA 90069, United States; Children’s Hospital of Philadelphia, 3401 Civic Center Blvd. Philadelphia, PA 19104, United States; Children’s Hospital of Philadelphia, 3401 Civic Center Blvd. Philadelphia, PA 19104, United States; Children’s Hospital of Philadelphia, 3401 Civic Center Blvd. Philadelphia, PA 19104, United States; Children’s Hospital of Philadelphia, 3401 Civic Center Blvd. Philadelphia, PA 19104, United States; University of Pennsylvania, 3400 Civic Center Boulevard Building 421, Philadelphia, PA 19104, United States; University of Texas Health Science Center at Houston, 7000 Fannin St. Houston, TX 77030, United States; University of Texas Health Science Center at Houston, 7000 Fannin St. Houston, TX 77030, United States; Dalian University of Technology, No. 2 Linggong Road, Ganjingzi District, Dalian, 116024, China; Dalian University of Technology, No. 2 Linggong Road, Ganjingzi District, Dalian, 116024, China; Dalian University of Technology, No. 2 Linggong Road, Ganjingzi District, Dalian, 116024, China; Dalian University of Technology, No. 2 Linggong Road, Ganjingzi District, Dalian, 116024, China; Department of Computer Science and Engineering, Korea University, 102 Woojung Hall of Informatics, 145 Anam-ro, Seongbuk-gu, Seoul, 02841, Republic of Korea; Department of Computer Science and Engineering, Korea University, 102 Woojung Hall of Informatics, 145 Anam-ro, Seongbuk-gu, Seoul, 02841, Republic of Korea; Department of Computer Science and Engineering, Korea University, 102 Woojung Hall of Informatics, 145 Anam-ro, Seongbuk-gu, Seoul, 02841, Republic of Korea; University of Nottingham, Nottingham NG7 2RD, United Kingdom; Imperial College London, Huxley Building, 180 Queen's Gate, South Kensington, London SW7 2AZ, United Kingdom; AIGEN Sciences, Office 507, 25 Ttukseom-ro 1-gil, Seongdong-gu, Seoul 04778, Republic of Korea; University of Edinburgh, 10 Crichton Street, Edinburgh, Scotland EH8 9AB, United Kingdom; Imperial College London, Huxley Building, 180 Queen's Gate, South Kensington, London SW7 2AZ, United Kingdom; Imperial College London, Huxley Building, 180 Queen's Gate, South Kensington, London SW7 2AZ, United Kingdom; University of Edinburgh, 10 Crichton Street, Edinburgh, Scotland EH8 9AB, United Kingdom; Department of Computer Science and Engineering, Korea University, 102 Woojung Hall of Informatics, 145 Anam-ro, Seongbuk-gu, Seoul, 02841, Republic of Korea; Department of Computer Science and Engineering, Korea University, 102 Woojung Hall of Informatics, 145 Anam-ro, Seongbuk-gu, Seoul, 02841, Republic of Korea; AIGEN Sciences, Office 507, 25 Ttukseom-ro 1-gil, Seongdong-gu, Seoul 04778, Republic of Korea; University of Manchester, 131 Princess Street, Manchester M1 7DN, United Kingdom; King Saud University, Riyadh 12371, Saudi Arabia; University of Manchester, 131 Princess Street, Manchester M1 7DN, United Kingdom; ASUS Intelligent Cloud Services, 10 Changi Business Park Central 2, #02-01 Hansapoint, Singapour 486030, Singapour; King Saud University, Riyadh 12371, Saudi Arabia; University of Manchester, 131 Princess Street, Manchester M1 7DN, United Kingdom; University of Manchester, 131 Princess Street, Manchester M1 7DN, United Kingdom; National Cheng Kung University, No. 1 Dasyue Road, East District, Tainan City, Taïwan 701, Taiwan; National Cheng Kung University, No. 1 Dasyue Road, East District, Tainan City, Taïwan 701, Taiwan; National Cheng Kung University, No. 1 Dasyue Road, East District, Tainan City, Taïwan 701, Taiwan; Children’s Hospital of Philadelphia, 3401 Civic Center Blvd. Philadelphia, PA 19104, United States; University of Pennsylvania, 3400 Civic Center Boulevard Building 421, Philadelphia, PA 19104, United States; Cedars-Sinai Medical Center, 700 N. San Vicente Blvd. Pacific Design Center, West Hollywood, CA 90069, United States

## Abstract

We present here an overview of the BioCreative VIII Task 3 competition, which called for the development of state-of-the-art approaches to automatic normalization of observations noted by physicians in dysmorphology physical examinations to the Human Phenotype Ontology (HPO). We made available for the task 3136 deidentified and manually annotated observations extracted from electronic health records of 1652 paediatric patients at the Children’s Hospital of Philadelphia. This task is challenging due to the discontinuous, overlapping, and descriptive mentions of the observations corresponding to HPO terms, severely limiting the performance of straightforward strict matching approaches. Ultimately, an effective automated solution to the task will facilitate computational analysis that could uncover novel correlations and patterns of observations in patients with rare genetic diseases, enhance our understanding of known genetic conditions, and even identify previously unrecognized conditions. A total of 20 teams registered, and 5 teams submitted their predictions. We summarize the corpus, the competing systems approaches, and their results. The top system used a pre-trained large language model and achieved a 0.82 F1 score, which is close to human performance, confirming the impact that recent advances in natural language processing can have on tasks such as this. The post-evaluation period of the challenge, at https://codalab.lisn.upsaclay.fr/competitions/11351, will be open for submissions for at least 18 months past the end of the competition. **Database URL:**  https://codalab.lisn.upsaclay.fr/competitions/11351

## Motivation

The field of clinical genetics focuses on identifying the genetic underpinnings of an individual’s medical presentation through the correlation of that individual’s genetic variants with their observable characteristics. These characteristics, which encompass an individual’s physical traits, physiological markers, and functional attributes, are known collectively as their ‘phenotype’. The dysmorphology physical examination is an essential part of clinical phenotyping and diagnostic assessment in clinical genetics. This examination involves documenting often subtle morphological variations in the patient’s facial features or body. It may also detect broader medical indicators, such as neurological dysfunction. The findings enable correlation of the patient with known rare genetic diseases. The dysmorphology assessments are routinely captured as unstructured free text in a listing of organ systems observations in the electronic health record (EHR), necessitating standardization for meaningful comparisons and downstream computational analysis. Genetics professionals often turn to the Human Phenotype Ontology (HPO), a specialized ontology designed for human genetics, for this purpose [1]. However, the manual coding of findings into HPO terms is labour-intensive and demands advanced genetics training to ensure coding accuracy. Recognizing the need for scalability and efficiency, there is a growing interest in automating this annotation process with natural language processing (NLP).

Advanced NLP methods can be used to retrieve and standardize phenotypic information from the unstructured free text of the dysmorphology physical exam at a large scale, thereby reducing the overhead associated with manual coding. However, several challenges must be addressed both for the extraction from the EHR and for the normalization to HPO terms. The extraction step challenges stem from the descriptive style, polarity of the findings (as findings during an examination may be normal or abnormal), and the disjoint or overlapping nature of these findings. The normalization process is difficult due to the vast scale and inconsistent levels of detail within the HPO ontology, resulting in the need to go beyond typical string matching strategies to efficiently handle variations in term detail and hierarchies. The BioCreative VIII Task 3 competition provided participants with an expertly annotated gold standard corpus from *actual* (de-identified) patient encounters, and challenged them to develop novel methods to overcome difficulties in extracting phenotypic descriptions from clinical notes and normalizing them to the most relevant HPO term using the latest advancements in NLP.

Advancements in NLP methods have been used to improve the performance of existing phenotype normalization systems over time. Two such systems, Doc2HPO [2] and Txt2hpo (https://github.com/GeneDx/txt2hpo/), use different approaches based on string matching. Txt2hpo matches stemmed terms in the input text to stemmed HPO terms, while Doc2HPO extends simple string matching, allowing users to choose from an ensemble of methods, including MetaMap and NCBO annotator, and creates a union of the results and presents them to the user for acceptance, edits, or rejection. Expanding beyond systems that use string matching, ontology or dictionary lookup, or rule-based engineering, Arbabi et al. [3] encoded the input text to vector representations using a convolutional neural network (CNN). Their system, Neural Concept Recognition, compared these representations to the embeddings from the ontology to identify relevant phrases. This model had some limitations, such as the inability to assess overlapping concepts. PhenoTagger [4] sought to address the limitations of Neural Concept Recognition by developing a system that combined dictionary matching with a BioBERT [5] model to identify HPO concepts, including overlapping terms, in text. To increase system speed, Feng et al. [6] used a two-level hierarchical CNN (TLH-CNN) before implementing a BERT model. This pipeline, named PhenoBERT, extracts relevant text using deep learning. These segments are first matched to HPO terms using dictionary-based matching; those not matched are processed by the TLH-CNN module to compute potential HPO terms, which are then input to the pretrained BioBERT model for final classification.

The BioCreative (Critical Assessment of Information Extraction Systems in Biology) shared task series is a community-driven initiative aimed at evaluating information extraction systems in the biological and medical domains. BioCreative was established to address the need for publicly available benchmark datasets and standardized evaluation criteria, ensuring fair comparisons across different natural language processing approaches [7]. Developed by biological database curators and domain experts, these datasets are publicly released to encourage the development of new applications and enhance existing ones. Since its inception in 2004, BioCreative Challenges have attracted an average of ~100 participants per event. BioCreative VIII, held in conjunction with the AMIA Annual Symposium in New Orleans, LA, USA, in 2023, featured four shared tasks. This paper presents the results of Track 3, which focused on evaluating current approaches for extracting and normalizing abnormal findings identified during dysmorphology physical examinations. Competing systems were required to extract spans referring to the findings and normalize them to HPO term IDs. This task is a particular instance of named entity recognition (NER), a fundamental challenge in NLP that requires systems to identify the main objects of discourse before extracting their relationship within specific events described in a document. By structuring unstructured data, NER is a crucial step in the automatic computation of the meaning of written documents, enabling fundamental processes such as literature mining, clinical decision support, and other applications [8].

## Task description and corpus

The goal of BioCreative VIII Task 3 was to identify, extract, and normalize findings noted in dysmorphology physical examination reports from clinical notes documented at the Children’s Hospital of Philadelphia (CHOP). These reports are structured as a series of observations, each beginning with a clear reference to the organ system being evaluated. Observations summarize notable findings documented by physicians during the examination in a few concise sentences. Findings have a polarity: *key findings* indicate abnormal phenotypes potentially suggestive of an underlying genetic condition, while *normal findings* describe observations that would be expected of a patient without a genetic condition. Normal findings are often explicitly noted to rule out specific conditions, confirming the health status of an organ system and facilitating communication with other healthcare providers. The task presented several challenges for automated information extraction system: extracting overlapping or disjointed findings, assessing their polarity (with normal findings to be ignored), and normalizing key findings to HPO terms, a difficult process due to the ontology’s extensive size (16 948 terms at the time of the competition).

Clinicians authored the dysmorphology physical examination reports using a specialized data entry form integrated into the EHR system, Epic Systems Inc. [9]. We structured the form into subsections, each dedicated to a specific organ system. Within each subsection, clinicians can input automatically calculated body measurements, select common key findings using predefined buttons, and supplement the report with free-text entries in the individual text box of the subsection. Once completed, the report is generated as a text document and can be incorporated into any clinical note. In April 2022, we collected all free-text entries, i.e. the observations, generated through this form. A total of 34 distinct clinicians contributed, each having authored at least 10 reports.

Our corpus includes 3136 free-text organ system observations from the dysmorphology physical examinations of 1652 patients evaluated at CHOP. Some observations occurred verbatim in multiple patients’ records, but these were combined into a single data point. We automatically de-identified the text of all observations using NLM Scrubber (https://lhncbc.nlm.nih.gov/scrubber/) and manually reviewed the text during the annotation process to preserve patient privacy. We sped up the annotation by pre-processing the observations with a baseline system, PhenoTagger [4]. Using a custom annotator interface (https://github.com/Ian-Campbell-Lab/HPO-Annotator), the annotators followed guidelines designed specifically for the purpose, identifying unambiguously documented key findings in the text *as well as* specifically mentioned normal findings. The identified findings were normalized to the closest unambiguous term included in the 2022-06-11 release of the HPO (https://github.com/obophenotype/human-phenotype-ontology/releases?page=2). Given the absence of terms for normal findings in the HPO, and to ensure their inclusion in the gold standard, annotators were instructed to normalize normal findings to the closest corresponding abnormal HPO term and mark them as negated. Four physicians and one medical student annotated the corpus. Our annotators double-annotated 890 observations, with an inter-annotator agreement of 0.844 average F1 score over all permutations between the annotators. We resolved discrepancies by selecting the annotations produced by the most senior clinician. Full details of the annotation process are reported in [10]. The corpus is freely available at https://github.com/Ian-Campbell-Lab/Clinical-Genetics-Training-Data/.

**Figure 1.**
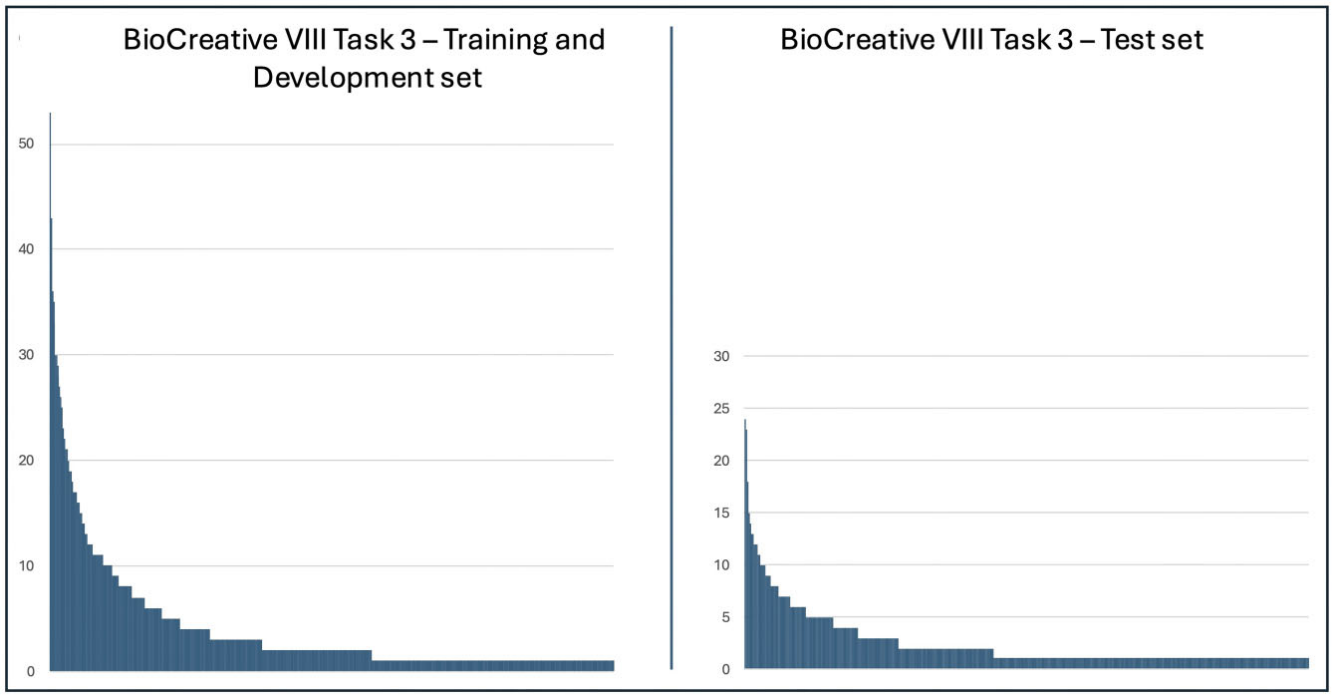
Frequency distributions of term mentions from the Human Phenotype Ontology in the BioCreative VIII Task 3 corpus.

Our corpus contains 934 HPO terms with a total of 4694 mentions, encompassing both key and normal findings. After removing normal findings, the training/validation set contains 702 HPO terms (2840 mentions), while the test set includes 498 terms (1258 mentions). The most frequent HPO term in both training/validation set and test set, is the term *HP:0100699—scarring*, with 53 mentions in the former and 24 in the latter. This is followed by the term *HP:0000218—high palate*, which has 43 mentions in the training and validation sets and 23 in the test set. Figure 1 illustrates the frequency distribution of the terms mentioned in the training/validation set and test set. Both distributions exhibit a pattern resembling Zipf’s distribution, with a small number of terms mentioned frequently and the majority infrequently. Specifically, only 11% (77 terms) occur more than 10 times in the training/validation set, and just 4% (18 terms) in the test set. We estimate our test set to be particularly challenging. It contains 135 HPO terms not found in either the training or validation sets, accounting for a total of 148 mentions, with the most frequent terms appearing only three times. Since 27% of the terms mentioned in the test set are absent from the training sets, supervised learning systems are required to generalize from their training to achieve perfect performance [11]. Furthermore, 55% of key findings in the test set—686 out of 1258—are expressed using phrases that differ from all preferred terms or their synonyms in the HPO, rendering strict exact-match approaches ineffective.

During the competition, our corpus was only released to registered participants; it is now publicly available. Each observation in the corpus contained (1) an observation ID uniquely identifying the observation; (2) the text of the observation starting with the organ system evaluated; (3) a term ID from the HPO ontology; (4) the start and stop positions of the span or spans of text denoting the finding; and (5) the polarity of the finding, *Negated* if the finding is normal or empty if the finding is abnormal. Examples of the annotated data are shown in Table 1.

**Table 1.** Examples of annotated observations with HPO terms, spans, and polarity—indicating a key finding if left empty, or a normal finding if marked as ‘Negated’.

ID	Text	HPO term	Spans	Polarity
1	EYES: partial synophrys, long lashes, horizontal slant	HP:0000664	14–23	
1	EYES: partial synophrys, long lashes, horizontal slant	HP:0000527	25–36	
2	MOUTH: normal lips, tongue, high palate	HP:0000218	28–39	
2	MOUTH: normal lips, tongue, high palate	HP:0000159	7–18	Negated
2	MOUTH: normal lips, tongue, high palate	HP:0000157	7–13, 20–26	Negated
3	NEUROLOGIC: very active	NA	NA	NA

Green highlights denote spans of key findings, while yellow highlights normal findings.

All special circumstances within the data and annotations were documented for the participants. Particularly, when observations mentioned two or more findings, the observation was repeated for each finding, with one finding annotated per repetition as illustrated in Table 1 for observations 1 and 2. Additionally, mentions of findings could span discontinuous segments of text. For these findings, we reported the start and end positions of each segment, separated by commas, in the order the segments appeared in the text, as shown for the discontinuous mention of the finding *normal tongue* in the third repetition of observation 2 in Table 1. The HPO ontology does not have terms to denote normal findings. Thus, as an alternative to this limitation, when possible, we normalized normal findings to the corresponding most specific abnormal term in the ontology and annotated it as *negated*; when it was not possible, i.e. no related terms were available to normalize the findings, we annotated the finding with not available. Of note, phenotypes encoded in the HPO that cannot be observed during a physical examination in a genetic encounter need not be considered by the normalizer. A list of these ‘non-observable’ terms in the HPO, deemed irrelevant to the task, was shared with the registered participants.

We randomly split the annotated corpus into three subsets: a training set (55%, 1716 observations), a validation set (15%, 454 observations), and a test set (30%, 966 observations). In addition, we added 2427 decoy observations to the test set, consisting of unannotated clinical observations collected from the EHR at the time we were preparing the datasets for the competition. The test set released to the participants contained only the observation IDs and the text of the observations.

In April 2023, the organizers released the training and validation sets to the registered participants. We released the test set on 15 tember 2023. We chose CodaLab Competitions [12], a free and open-source web-based platform, to host the competition. Participants were allotted 3 days to automatically predict the extraction and normalization of spans in the test set. While we cannot entirely rule out the possibility of participants manually correcting their predictions before submission, if any corrections were attempted, the large number of decoy observations in the test set and the strict 3-day submission window should have limited their number and their impact on the overall performance of the systems. Participants were required to submit their predictions by 18 September 2023, online to the Task 3 competition site on CodaLab (https://codalab.lisn.upsaclay.fr/competitions/11351). Each submission triggers an automated script to evaluate the submitted system’s predictions against our labelled test set. The test set was also uploaded to CodaLab but hidden from participants. Thus, the participants can see their results instantaneously, but not how they compare to those of others: We hid the leaderboard in CodaLab until the official release of the results during the BioCreative VIII workshop (https://www.ncbi.nlm.nih.gov/research/bionlp/biocreative#bioc-8). We limited each participating team to a maximum of three different system prediction submissions.

The use of CodaLab not only eliminated the need for the manual management of participants’ submissions and results but also allowed us to keep the competition open beyond the BioCreative event. This means that any researcher can register on CodaLab for access to the data—i.e. the labelled training and validation sets and the unlabelled test set, including the decoy observations—and submit their system’s predictions on the test set to the competition for evaluation for at least 18 months after the competition. Their results will be evaluated using the same evaluation script, against the same test data thus ensuring an equitable comparison against all previously evaluated systems.

## Evaluation

### Metrics

We evaluated each competing systems against two subtasks of the overall task. For subtask A, we evaluated the ability of the competing systems to normalize to HPO terms all mentions of key findings in an observation (*Normalization-only*), regardless of whether they could detect the spans of the mentions. We selected the standard precision, recall, and F1 scores to measure their performance on subtask A. A true positive (TP) is a correctly predicted HPO term for a key finding in an observation. That is a predicted HPO term for an observation exactly matching one of the annotated HPO terms for that observation in the gold standard. A false positive (FP) is a predicted HPO term that does not exactly match one of the HPO terms in the gold standard for that observation. A false negative (FN) is an HPO term that was present for an observation in the gold standard but was missed by the system. The precision (P) is the ratio of all correct HPO term predictions (TP) to all HPO terms predicted by the system (TP + FP), equation (1). The recall (R) is the ratio of all correct HPO term predictions (TP) to all HPO terms in the gold standard (TP + FN), equation (2). The F1 score (equation (3)), which was used to summarize the overall performance of the system, is the harmonic mean of P and R.


(1) \begin{eqnarray*}
{\mathrm{P}} = {\mathrm{TP}}/\left( {{\mathrm{TP}} + {\mathrm{FP}}} \right),
\end{eqnarray*}

(2) \begin{eqnarray*}
{\mathrm{R}} = {\mathrm{TP}}/\left( {{\mathrm{TP}} + {\mathrm{FN}}} \right),
\end{eqnarray*}

(3) \begin{eqnarray*}
{\mathrm{F}}1 = 2^*\ \left( {{\mathrm{P}} ^*{\mathrm{R}}} \right)/\left( {{\mathrm{P}} + {\mathrm{R}}} \right).
\end{eqnarray*}

In subtask B, we evaluated the system’s ability to both detect the spans of mentions of key findings and normalize them as a supplementary evaluation (*Overlapping Extraction and Normalization*). We selected the overlapping precision, recall, and F1 scores as the metric to evaluate subtask B. For overlapping extractions, the system was rewarded when it extracted the spans or a part of the spans of a labelled key finding mention and correctly assigned the labelled HPO term ID to the mention. For example, in Table 1, a key finding is ‘*high palate* (span 28–39)—HPO ID: 0000218’. If the system predicted ‘*palate* (span 33–39)—HPO ID: 0000218’, it would be scored as a TP because ‘*palate*’ (span 28–39) is a substring of ‘*high palate*’ (span 33–39). Note, however, that the predicted HPO terms were required to match exactly the HPO terms in the gold standard for the mentions of the key findings; if the system predicted a different HPO term on the overlapping span, the prediction would be scored as an FP. In the competition, we chose the best system to be the system, which achieved the best F1 score on the Normalization-only evaluation. This subtask and metric were chosen based on the real-world application of the system where only the normalization of key findings in observations is medically relevant to physicians. We computed all metrics at the level of individual observations.

### Baseline systems

Multiple baseline systems, freely available and open source, were available to participants to perform our task off the shelf. We evaluated *txt2hpo* (https://github.com/GeneDx/txt2hpo/), *Doc2HPO* [2], *NeuralCR* [3], *PhenoBERT* [6], and *PhenoTagger* [4]. A brief overview of these systems is reported in the ‘Introduction’ section. We ran and evaluated these systems on our gold standard test set without further training. PhenoTagger was the best-performing system with an F1 score of 0.633 when performing the normalization-only subtask, which we reported in Table 2. The authors of PhenoTagger approached the problem with a method that combines dictionary matching with machine learning. The authors compiled a dictionary from the list of all observable terms and their synonyms in the HPO. They used this dictionary to build a distantly supervised training dataset. They trained a BioBERT model to classify each *n*-gram to an HPO term ID or the special tag ‘None’ while retaining terms surpassing a predetermined threshold. The outcomes of the dictionary matching and the classifier were subsequently combined to generate the final predictions.

**Table 2.** Systems performance (F1: F1 scores; P: precision; R: recall) and system summaries (TG: term generation; TE: term extraction; TN: term normalization). The highest scores are highlighted in bold.

	Normalization-only			Overlapping extraction and normalization			
Team	F1	P	R	F1	P	R	System synopsis
Soysal and Roberts [13]	**0.820**	**0.842**	**0.799**	**0.817**	**0.841**	**0.794**	TG: ChatGPT + TN: exact matching on stems
Qi et al. [14]	0.763	0.831	0.706	0.762	0.830	0.704	TE: multiple W^2^NER instances relying on various BERT models + TN: ensemble of Bioformer and dictionary matching
Kim et al. [15]	0.745	0.735	0.755	0.743	0.734	0.752	TG: fined tuned ChatGPT + TN: synonym marginalization (BioSyn)
Alhassan et al. [16]	0.723	0.718	0.728	0.721	0.717	0.726	TG: FLAN-T5-XL fined tuned with LoRA + TN: distance similarity (RoBERTa) and candidates re-ranking with sentence transformer cross-encoder
Lin et al. [17]	0.644	0.762	0.557	0.642	0.761	0.556	TN: ensemble (PhenoTagger and PhenoBERT) + TE: BioLinkBERT
Baseline [4]	0.633	0.587	0.687	0.632	0.586	0.685	Ensemble of a BioBERT multi-class classifier and dictionary matching

### Systems

#### Results

Among the 20 teams that registered for the shared task, 5 participated in the end, and submitted 3 prediction files each (the maximum number of submission files authorized). We kept the best predictions for each team. We present the results of each team and a brief synopsis of the architecture of their best approach in Table 2. All systems outperformed the best-performing baseline system, PhenoTagger. Based on a large generative model for the extraction and a combination of generation and dictionary matching for the normalization, the best system [13] achieved an F1 score of 0.82, only 2 points under human performance on this task (i.e. the inter-annotator agreement of 0.844 average F1 score). This confirms the recent technical improvement made possible by large language models (LLMs).

#### Individual system descriptions

The five teams who submitted predictions of their systems for evaluation against the gold standard were invited to submit a technical summary describing in detail their approach for creating their system(s) for the BioCreative VIII Task 3 competition. Table 3 presents a summary of the teams that participated. The individual systems descriptions are presented next in rank order of their results in the competition, summarized from their submitted technical abstract [13, 14, 15, 16, 17].

**Table 3.** Summary of participating teams.

Team	Institution	Country	System description paper
1	The University of Texas Health Science Center at Houston	USA	Soysal and Roberts [13]
2	Dalian University of Technology	China	Qi et al. [14]
3	Korea University, Imperial College, AIGEN Sciences, University of Edinburgh, University of Nottingham	Korea, UK	Kim et al. [15]
4	University of Manchester, King Saud University, ASUS	United Kingdom, Saudi Arabia, Singapore	Alhassan et al. [16]
5	National Cheng Kung University	Taiwan	Lin et al. [17]

#### Team 1: The University of Texas Health Science Center at Houston

*Summary: Term extraction was performed using the generative model ChatGPT with few-shot learning to identify the spans of key findings. Term normalization was first attempted through dictionary matching on the extracted spans. If no match was found, dictionary matching was then applied to the corresponding HPO-preferred terms generated by ChatGPT*.

We used, at the time of writing, the latest version of OpenAI’s LLM, GPT-4 [18], to solve the problem of extracting key findings from given observations and normalizing them to concepts in the HPO. Prior to model training, we performed preprocessing steps. First, we reviewed the annotations in the training and validation sets and corrected any inconsistencies in the annotation. We corrected inconsistencies in the spans selected for HPO terms, improving consistency in the spans would improve performance in span selection. To increase performance in HPO concept selection for a given entity, we also corrected inconsistencies in selected HPO terms in the annotations that may arise when there are similar terms that all closely match a selected entity. Our second preprocessing step was to remove all normal finding concept annotations from the training and validation sets.

Using OpenAI’s ChatCompletion API (https://platform.openai.com/docs/api-reference/chat) with the GPT-4 model, we prompted the model to extract key findings from a given text and return an answer with two elements: (i) the HPO term and (ii) the original text marked with brackets around the words associated with the HPO term. We used the bracketed text to identify the character offsets for each entity, allowing for the extraction of continuous or disjoint entity spans. We used this approach to overcome the shortcomings in GPT-4’s ability to identify character offsets. Next, we experimented with a few-shot learning approach where we provided examples with each request to better convey our intent to GPT-4. For each request, we added 25 examples generated specifically for that request. The first 15 examples were selected from the training data based on them being most similar to the observation text and included at least one observation annotation. Similarity was scored using spaCy document similarity [19], which performs cosine similarity using an average of word vectors. Five manually selected ‘tricky’ examples were added to guide GPT-4 through task requirements that were not always followed as described in the prompt, and the final five were negative examples that contained no key finding to convey to GPT-4 that empty responses were allowable.

For entity normalization, we built a dictionary of HPO terms, including the preferred terms, synonyms, and the labelled spans in the annotated observations, that map to HPO IDs. All terms in the dictionary were stemmed to facilitate exact matching. We used two different matching algorithms to normalize the extracted named entities. In the first matching algorithm, the first part of the answer, the HPO term identified by GPT-4 for an observation, is ignored. The second part of the answer, text marked, is stemmed and matched with the entries of the dictionary. The second matching algorithm follows the steps of the first; however, if no match is found in the dictionary after the search, the HPO term identified by GPT-4 is stemmed and that term is then searched for in the dictionary.

We submitted two runs for evaluations; the first run used GPT-4 with few-shot learning, and our first matching algorithm and the second run used GPT-4 with few-shot learning and our second matching algorithm. Our second run, using both the extracted entity and the GPT-4 identified HPO term for normalization, achieved the highest F1-score of 0.8197 for normalization-only and 0.8168 for overlapping extraction and normalization. We found that our approach with few-shot learning where GPT-4 was shown examples of expected responses significantly improved performance over trying to elicit the same response behaviours from prompting alone. The system is available at https://github.com/esoysal/phenormgpt.

#### Team 2: Dalian University of Technology

*Summary: Term extraction was performed using the W^2^NER architecture instantiated with various BERT-based classifiers working in parallel to identify spans of all findings. All extracted candidates were normalized using the classifier Bioformer, with additional candidates retrieved through dictionary matching. Only the most likely candidates were passed to a voting ensemble to select their final HPO IDs. Postprocessing rules were applied to remove overlapping candidates and normal findings*.

To automatically extract and normalize key findings from organ system observations, we employed a deep learning-based pipeline approach that divided the process into two subtasks: NER and named entity normalization (NEN). This approach was based on our prior work, PhenoTagger [3]. For the phenotype entity recognition part, we sought to extend the baseline system PhenoTagger, which demonstrates competency in the recognition of contiguous phenotype entities but has limitations in effectively identifying discontinuous entities. We addressed this challenge by leveraging the W^2^NER system [20], which reframes the NER task into predicting relationship categories between word pairs. We tried Bioformer [21], BioBERT [5], BioLinkBERTlarge [22], Biom-ELECTRAlarge [23], Clinical BERT [24], PubMedBERT [25], and Clinical PubMedBERT [26] models for W^2^NER, including the large versions of both BioBERT and PubMedBERT. The models were trained using the training set from the competition. For entity normalization, we used the deep learning-based classification method from PhenoTagger to classify the NER results to a specific HPO term. We experimented with Bioformer, BioBERT, and PubMedBERT, choosing Bioformer as the final classification model based on the results on the validation set (F1 scores of 0.7165, 0.7142, and 0.7140, respectively). After passing through Bioformer, candidate entities are classified by the softmax layer, which outputs a probability score. We manually set a threshold, keeping only the results with a probability above this threshold. We experimented with two thresholds to generate the NEN results: 0.8 and 0.95. On the validation set, the model had a higher recall rate at a threshold set to 0.8 and higher accuracy at a threshold of 0.95. Generally, when evaluating the performance of our system on the validation set across identical indicators, a threshold set at 0.8 yielded a better F1 score.

An additional voting ensemble method for entity recognition was created using the nine distinct models where a threshold, *m*, was set and the entity extracted by more than *m* models was selected as the ensemble result. We ran experiments with *m* = 2, 3, 4, and 5, achieving the best results with *m* = 2. We applied post-processing rules to the ensemble results to remove overlapping recognition results and normal findings. Finally, entities identified using the dictionary-based part of PhenoTagger were added as supplements to the results. On the validation set, the ensemble achieved the best result on the normalization-only task with an F1 score of 0.7975. The highest scoring singular model on normalization-only was the W^2^NER (PubMedBERTlarge) with PhenoTagger achieving an F1 score of 0.7642, a 4.45% increase in performance over PhenoTagger alone. For the entity extraction and normalization task, this model had a 6.63% performance increase over PhenoTagger (F1: 0.7317 vs. 0.6654), although as before, the ensemble method achieved the highest overall score with an F1 score of 0.7502.

For the final submission, the highest scoring run for normalization was the model trained on the training and validation sets, with the NEN threshold set to 0.95 to generate the final results. The model achieved an F1 score of 0.7632 on the official test set.

#### Team 3: Korea University/Imperial College/AIGEN Sciences/University of Edinburgh/University of Nottingham

*Summary: Term extraction was performed using a generative fine-tuned ChatGPT model to identify spans of all findings and subsequently class them as normal or key findings. Term normalization was conducted with the classifier BioSyn, trained using synonym marginalization on the shared task training set*.

We developed a pipeline consisting of two parts. In the first part, we aimed to identify HPO entities present in the input text using NER. We first identified four edge cases in the training data: observations with discontinuous findings, observations with only continuous findings, observations with normal findings, and observations with no findings. We split the set into 70/30 training/validation sets, keeping an even proportions of edge cases in each set. We assessed multiple models that have shown state-of-the-art performance in both continuous and discontinuous NER. We chose ChatGPT and W^2^NER, which outperformed other competing systems. We constructed our first NER model using the ChatGPT Finetuning API (https://platform.openai.com/docs/guides/model-optimization). The data were preprocessed to expand abbreviations and translate symbols into text format. We performed the extraction in two steps using the fine-tuned ChatGPT. In the first step, we extracted all findings, whether key or normal findings. In the second step, we classify the extracted findings into the two categories. Our second NER model used the W^2^NER architecture, which we optimized by fine-tuning the hyperparameter. We evaluated several BERT models to use as the first layer of the system, including BioBERT [5], SciBERT [27], PubMedBERT [25], and ClinicalBERT [28]. We found that ClinicalBERT performed best. We trained W^2^NER to extract only key findings.

For the second part of our pipeline, the NEN, we used a combination of methods. To incorporate more synonyms and create a generalizable dictionary, we flattened the HPO dictionary and removed unused terms. We used SapBERT [29] for embeddings. As it was pre-trained on UMLS data, we undertook pre-finetuning of SapBERT using our dictionary. We call this pre-fine-tuned model, PhenoSapBERT. To enhance our research, we used the synonym marginalization method, BioSyn [30], which uses an iterative candidate retrieval method and additive synonym incorporation during marginalization.

For our submissions, we used models with the fine-tuned ChatGPT, W^2^NER, and an ensemble of the two for NER. All models used BioSyn for NEN. The best model was the fine-tuned ChatGPT model, which achieved an F1 score of 0.7448 for normalization-only and an F1 score of 0.7428 for overlapping extraction and normalization.

#### Team 4: University of Manchester/King Saud University/ASUS

*Summary: Term extraction was performed using a generative fine-tuned T5 model to identify the spans of key findings. For normalization, RoBERTa was used to embed the identified key findings alongside all HPO terms. The 30 most similar HPO terms were then scored using a sentence transformer cross-encoder, and the top-ranked terms were selected as their corresponding HPO terms*.

For BioCreative VIII Task 3, we developed DiscHPO, a two-component pipeline. The first component detects continuous and discontinuous named entity spans, and the second normalizes the extracted spans to associated HPO identifiers. We framed the NER problem as a sequence-to-sequence problem [31]. In our preprocessing, we converted the numeric span offsets in the training data to the actual word span prefixed with the entity type: key finding or normal finding.

For the NER component in our pipeline, we investigated several variants of the Text-to-Text Transfer Transformer (T5) [32] to create a sentence-level NER module based on fine-tuning a pre-trained sequence-to-sequence encoder–decoder language model. We examined several T5 architectures, including the original T5, Flan-T5 [33], and SCIFIVE [34]. For optimizing the Flan-T5-XL architecture, we used low-rank adaptation (LoRA) [35], a parameter-efficient fine-tuning approach.

For the normalization component, we fine-tuned the all-roberta-large-v1 model (https://huggingface.co/sentence-transformers/all-roberta-large-v1) to create embeddings for both HPO terms in the ontology and the extracted entity spans from the observations. We excluded the HPO terms that were in the list of excluded HPO terms provided by the task organizers. To train on semantic similarity, pairs of spans from the training set and their HPO terms were used for fine-tuning. Then, to identify relevant candidates, we compared the embeddings representing the spans with those representing the HPO terms. We performed semantic matching with cosine similarity. The top 30 matches based on semantic similarity were then passed to a sentence transformer cross-encoder model, ms-marco-electra-base (https://huggingface.co/cross-encoder/ms-marco-electra-base), which re-ranks the candidate HPO terms by calculating the scores for each span-candidate term combination. After the results were sorted, we selected the top-scoring match as the final output.

We evaluated the different T5 models, with the normalization module, on the validation set using the evaluation script provided by the task organizers. We obtained the best results on this dataset with FlanT5-XL with LoRA with an F1 score of 0.742 for normalization only, and an F1 score of 0.738 for overlapping extraction and normalization. For our submitted final runs, we used the model with this architecture, varying the alpha hyperparameter of LoRA to either 512 or 1024 and trained our model either using only the training dataset or combining the training set with the validation set. The normalization component was unchanged in all runs. We achieved our highest score on the official test set using FlanT5-XL with LoRA α = 512, trained solely on the training set, resulting in an F1 score of 0.7220.

#### Team 5: National Cheng Kung University

*Summary: The focus was on improving term extraction from existing baselines, PhenoTagger and PhenoBERT, which cannot process discontinuous findings. These baselines first extracted and normalized findings. A fine-tuned sequence labeller, BioLinkBERT, was then used to discard normal findings and retrieve spans of key findings from the observations*.

We concentrated on the extraction and normalization subtask due to the limitation of existing methods, which can only identify consecutive spans of HPO terms. In the training and validations sets, 14.4% and 14%, respectively, are annotated as discontinuous spans, implying that these methods would be likely to miss these observations. To improve upon the performance of the baseline systems, we propose a sequence tagging framework. We preprocessed the observations, first by tokenizing them using the ‘word_tokenize’ function of NLTK [36]. Each token was then labelled for sequence tagging using a tagging schema for each token as either key finding, normal finding, or other. For sequence-level training, we leverage the HPO dictionary provided by the organizers. We then append the associated HPO text (also tokenized by NLTK) to the observation, with an insertion of the [SEP] token to separate the two types of text. We fine-tuned BERT for sequence tagging, testing our approach with PubMedBERT [25], BioLinkBERT [22], and Bioformer [21].

To obtain the predictions for Task 3, we structured three main steps in our pipeline: (1) We employed base models, such as PhenoTagger [4] or PhenoBERT [6], to generate a preliminary prediction set of HPO terms from the observations in the evaluation set; (2) we performed span localization based on the preliminary prediction set and appended each HPO term of the preliminary prediction set to an observation with a [SEP] token and then used our trained sequence tagging model to predict the labels of the input tokens; and (3) we aggregated the subword tokens from the BERT output and obtained the HPO positions corresponding to the tokens predicted as key findings. HPO terms predicted as ‘normal’ by our sequencing tagging model were filtered out before obtaining the final prediction. For the normalization-only task, our systems achieved an F1 score of 0.644 on the official test set. Our model did improve exact span localization over the base models, implying that these methods could be used on top of other approaches to enhance span localization capabilities.

## Discussion

All systems proposed a common, yet effective, pipeline approach that divides the process into two subtasks: extraction followed by normalization [37]. The teams adopted two different strategies to handle discontinued and overlapping terms, which accounted for 16.9% (213/1258) of the terms in our test set. The first strategy unifies the extraction of all terms (continuous and discontinuous) by identifying relations between the tokens. This strategy was successfully implemented by Qi et al. [14] with an ensemble of W^2^NER [20]. The second strategy leverages recent advancements in generative models to reframe the standard NLP task of entity extraction as a straightforward question-answering task [38]. Teams adopting this strategy prompted an LLM to identify and list all HPO terms mentioned in an observation, requesting the LLM to format its response according to a predefined structure such as tabular or JSON formats, thus enabling the automatic retrieval of spans corresponding to the mentions [8]. This strategy was the most popular on our task with three of the five competing systems implementing it [13, 15, 16]. All systems explicitly handled the detection of normal findings by detecting negations in the context of the terms extracted or by training dedicated classifiers. We previously found that explicitly handling normal findings in this way is very effective since our transformer-based model [10], trained in a similar way, identified almost perfectly the normal findings in the test set.

The participants of the shared task employed all currently known approaches to normalize the terms, i.e. extraction-based, retrieval-based, and generative-based approaches, making their methodologies representative of the state-of-the-art in the field [39]. The intuitive exact matching approach selects the HPO term that matches exactly a key finding extracted in an observation. It was the default approach for three out of five systems; however, due to its obvious limitation of normalizing only matching candidates, it was combined by all three with machine learning to process the remaining cases.

All but one system normalized those cases not handled by exact matching using standard retrieval-based approaches [40], either by predicting the most likely HPO term with a multi-class classifier [14, 17] or by selecting the most similar HPO term to the candidate within an embedding space [15, 16]. Both approaches have well-known limitations. First, multi-class classification struggles with limited generalization and scalability, relying heavily on their training data and making them ineffective at handling rare or unseen HPO terms during training. This limitation becomes even more pronounced as the number of possible target terms increases, as when normalizing to large ontologies like the HPO, making the training of such systems with multi-class classification increasingly difficult as more data would need to be annotated to improve their performance [40]. On the other hand, clustering similar HPO term-candidate pairs faces challenges due to impoverished semantic representation: These models rely on computing meaningful representation of both the candidate phrase and its corresponding HPO term to assess similarity accurately. However, this computation may be difficult for several reasons: The candidate may be described rather than explicitly named or contain out-of-vocabulary tokens, such as misspellings; HPO terms, while typically defined in the ontology, lack real-world usage examples; and contextual understanding remains limited, as incorporating all relevant information from the surrounding sentence remains a challenge—one that only the largest language models have recently begun to address properly [41].

The most innovative and successful system in the task [13] leveraged the capabilities of LLMs for normalization. The authors employed prompt engineering to generate HPO terms corresponding to extracted candidates using the most advanced LLM available at the time of the competition. While this approach was conceptually simple, it proved highly effective by avoiding common pitfalls and maximizing the strengths of LLMs. To minimize hallucinations—such as mapping an HPO term to a phrase not found in the observation text—the system combines extraction and normalization within a single prompt, reducing the risk of generating inaccurate or fabricated information. This approach forced the LLM to first tag the candidate to normalize within the text of the observation before generating the associated HPO term, effectively anchoring its decision in context. Additionally, instead of prompting the LLM to directly generate HPO term IDs—which LLMs empirically struggle with [42]—the authors instructed the model to generate the preferred HPO term, for which the LLM was more likely to have an internal representation. It is uncertain whether the HPO (or a part of it, such as terms definitions) was explicitly included in the pretraining corpus of the proprietary LLM used by this team (ChatGPT-4). However, the model had clearly acquired knowledge of most, if not all, HPO terms—either directly or indirectly—through public sources such as scientific literature or discussion. Growing evidence suggests that LLMs develop internal representations of real-world entities and their relationships [43] allowing them to move beyond mere memorization of training data. Instead, they generalize by inferring new facts and integrating them into their reasoning. We hypothesize that LLMs maintain an internal representation of most HPO terms, which they utilize to compute a meaningful similarity score between the labelled HPO term and its candidate mention, understanding the latter in the context of the observation. This ability likely contributes to the effectiveness of LLM-based normalization approaches to solve the task.

Despite the advantages of LLM-based normalization, the best-performing system [13] is not flawless, and certain observations remain challenging. We analysed 105 errors across 83 randomly selected observations. Among these, 36 were FNs, where the system failed to detect HPO terms explicitly mentioned in the text. 24 were FPs, in which the system mistakenly identified normal findings as key findings. The remaining 45 errors involved cases where the system correctly identified the relevant spans—or portions of them—but assigned incorrect HPO terms.

We summarize the error categories and provide examples in Table 4. Through manual analysis, we identified 10 non-exclusive error categories. Notably, two categories (lines a and b in Table 4) account for a third of all errors. The most frequent errors (a. *n* = 21) are cases where the model retrieves hypernyms of the annotated HPO terms. While these terms are more general than those selected by clinicians, they remain accurate and closely related, making them acceptable in certain use cases. The next most common errors (b. *n* = 18) arise from descriptive observations, where clinicians describe findings rather than using specific medical terms present in the HPO. These descriptions often lack lexical overlap with the preferred terms or their synonyms, making them challenging for the model to normalize. Despite the generalization capabilities of LLMs, they still appear to rely, at least partially, on keyword matching to retrieve the appropriate HPO terms.

**Table 4.** Error categories for the winning system.

	Error type	Count	Example
a.	Hypernym predicted	20% [21]	HANDS FEET: *hypoplastic 3rd toe digits* bilaterally [*Short 3rd toe*—HP:0005 643 labelled, *short toe*—HP:0001 831 predicted]
b.	Descriptive gold true span	17.1% [18]	EXTREMITIES: *wind swept hands* [*Ulnar deviation of finger—*HP:0009 465 labelled, missed prediction]
c.	Misplaced attention	8.6% [9]	NEUROLOGIC: abnormal *gait, wide based* [*Broad-based gait*—HP:0002 136 labelled, *Gait disturbance*—HP:0001 288 predicted]
d.	Complex mention requiring inference	6.7% [7]	EYES: subjectively narrow palpebral fissures, *horizontal eyebrows* [*Horizontal eyebrows*—HP:0011 228 labelled, missed prediction]
e.	Imperfect ontology	4.8% [5]	GENITALIA: *enlarged scrotum*; no hernia palpated [*Abnormal scrotum morphology*—*HP:0 000045* labelled, missed prediction]
f.	Misspelling	2.9% [3]	HANDS FEET: clinodactyly, *index fingers curived* [*Clinodactyly of the 2nd finger*—HP:0040022 labelled, missed prediction]
g.	Negation unsolved	2.9% [3]	EXTREMITIES: *unable to hyperextend elbows* and knees beyone 10 degrees [*Limited elbow extension*—HP:0001 377 predicted]
h.	Unknown reason	6.7% [7]	CHEST: thorax asymmetry. Pectus carinatum [*Asymmetry of the thorax*—HP:0001 555 labelled, missed prediction]
i.	Annotation error	19% [20]	–
j.	Contentious annotation	11.4% [12]	–
	Total	105	

Less frequent errors (c. *n* = 9) result from a shift in the model’s attention, causing it to focus on phrases outside the correct annotation span and to overemphasize unrelated words. For instance, in the observation *abnormal gait, wide-based*, the model focused on the word *abnormal*, leading it to incorrectly predict *Gait disturbance (HP:0001 288)*. Other infrequent errors (d. *n* = 5) occur when term normalization requires logical inference. For example, while *horizontal eyebrows* may not typically indicate an abnormality, within a physical exam context, it signals a key finding corresponding to *Horizontal eyebrows (HP:0011 228)*. The remaining error categories each account for less than 5% of errors.

Despite our best efforts, we discovered several annotation errors while reviewing discrepancies between the model’s output and the gold standard. Specifically, 12 annotation errors (line j in Table 4) involved terms such as *Scarring (HP:0100699)* or *Tube feeding (HP:0033 454)*, which were inconsistently annotated. Since these terms may not be indicative of genetic conditions in some contexts, they could arguably be disregarded. The 20 annotation errors (line i) were outright annotation mistakes, highlighting the inherent complexity of this task. We will upload an updated version of the test set on CodaLab as soon as it becomes available.

## Conclusion

In this paper, we presented the results of the BioCreative VIII Task 3, which challenges participants to extract and normalize key findings in 3136 observations from dysmorphic examinations. Given an observation, the task consists of detecting the spans of all key findings mentioned and returning the list of their corresponding IDs in the HPO. All five competing systems addressed the task using a pipeline architecture, where key findings were extracted first, followed by independent normalization. Most systems (Teams 1, 3, and 4) prompted LLMs to list the spans of key findings in observations. Participants used a wider range of methods for normalization. All started with the default exact-matching approach, which paired obvious candidates with their corresponding HPO terms. For more complex candidates, machine learning was employed, ranging from prompting LLMs to suggest their corresponding HPO preferred terms (Team 1), to identifying their closest HPO term in embedding spaces (Teams 3 and 4), or using conventional multi-class classifiers (Teams 2 and 5). The top-performing system (Team 1) achieved 0.82 F1 score when normalizing the terms, which very closely matches human performance. However, this high performance is based on a relatively straightforward dataset of dysmorphic examination observations, characterized by well-structured, short sentences with clearly defined organs, which we manually extracted from the EHR. The broader task of detecting and normalizing HPO terms in *general* EHR notes is significantly more challenging, as indicated by our preliminary experiments, which show a notable drop in performance on these less structured and more complex mentions.
